# *N*-{2-[2-(2-Cyano-4,6-di­nitro­phen­yl)diazen­yl]-5-(diethyl­amino)phen­yl}acetamide

**DOI:** 10.1107/S160053681203992X

**Published:** 2012-09-26

**Authors:** Lihua Lu

**Affiliations:** a College of Chemistry and Pharmaceutical Sciences, Qingdao Agricultural University, Qingdao 266109, People’s Republic of China

## Abstract

The title compound, C_19_H_19_N_7_O_5_, exhibits substitutional disorder of the *ortho*-nitro and cyano groups, with site-occupancy factors of 0.686 (7):0.314 (7). The two aromatic rings are essentially coplanar, with a dihedral angle of 6.6 (5)°. In the diethyl­amino group, the two ethyl groups lie on the same side of the amino­benzene plane. An intra­molecular N—H⋯N hydrogen bond links the amino and diazenyl groups.

## Related literature

For the influence of the substitutent on molecular planarity, see: Freeman *et al.* (1997 ▶); Lu & He (2012 ▶). For similar structures, see: Gong & Lu (2011 ▶); He *et al.* (2009 ▶). 
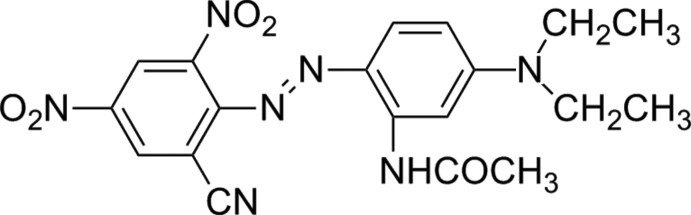


## Experimental

### 

#### Crystal data

C_19_H_19_N_7_O_5_
*M* *_r_* = 425.41
Monoclinic, 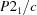

*a* = 5.0995 (5) Å
*b* = 30.792 (3) Å
*c* = 12.7211 (11) Å
β = 93.569 (1)°
*V* = 1993.6 (3) Å^3^
*Z* = 4
Mo *K*α radiation
μ = 0.11 mm^−1^
*T* = 298 K
0.37 × 0.11 × 0.07 mm

#### Data collection

Bruker SMART CCD area-detector diffractometer
Absorption correction: multi-scan (*SADABS*; Sheldrick, 1996 ▶) *T*_min_ = 0.962, *T*_max_ = 0.993
10384 measured reflections
3487 independent reflections
1453 reflections with *I* > 2σ(*I*)
*R*_int_ = 0.108

#### Refinement

*R*[*F* ^2^ > 2σ(*F* ^2^)] = 0.067
*wR*(*F* ^2^) = 0.157
*S* = 1.00
3487 reflections
311 parameters
H-atom parameters constrained
Δρ_max_ = 0.20 e Å^−3^
Δρ_min_ = −0.19 e Å^−3^

### 

Data collection: *SMART* (Siemens, 1996 ▶); cell refinement: *SAINT* (Siemens, 1996 ▶); data reduction: *SAINT*; program(s) used to solve structure: *SHELXS97* (Sheldrick, 2008 ▶); program(s) used to refine structure: *SHELXL97* (Sheldrick, 2008 ▶); molecular graphics: *ORTEP-3* (Farrugia, 1997 ▶); software used to prepare material for publication: *SHELXTL* (Sheldrick, 2008 ▶).

## Supplementary Material

Crystal structure: contains datablock(s) I, global. DOI: 10.1107/S160053681203992X/aa2072sup1.cif

Structure factors: contains datablock(s) I. DOI: 10.1107/S160053681203992X/aa2072Isup2.hkl

Supplementary material file. DOI: 10.1107/S160053681203992X/aa2072Isup3.cml

Additional supplementary materials:  crystallographic information; 3D view; checkCIF report

## Figures and Tables

**Table 1:** Hydrogen-bond geometry (Å, °)

*D*—H⋯*A*	*D*—H	H⋯*A*	*D*⋯*A*	*D*—H⋯*A*
N3—H3⋯N2	0.86	2.01	2.676 (4)	133

**Fig. 1.**
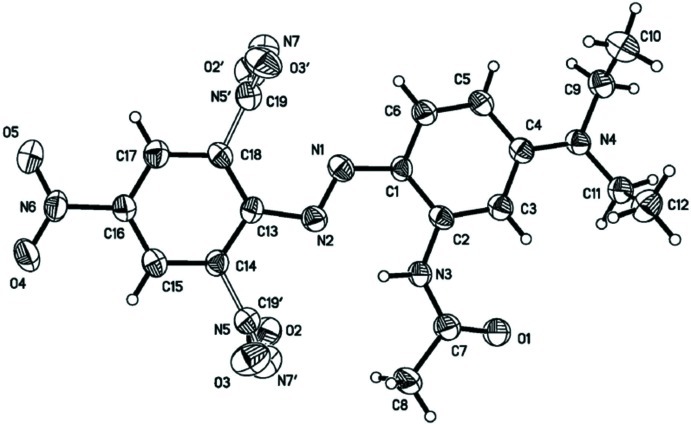
ORTEP drawing of 2-acetylamino-4-(N,N-diethylamino)-2',4'-dinitro-6'-cyanoazobenzene.

## supplementary crystallographic information

### Comment

It is well known that technical properties and functionalities of chemcial compounds are often related to their molecular structures and their inter/intra-molecular interactions in crystalline state. Hence, the structural investigation of different compounds in solid state is very important.

The title compound, C_19_H_19_N_7_O_5_, exhibits substitutional disorder of the nitro group at 2'-position and the cyano group at 6'-position, with site-occupancy factors of 0.686 (7): 0.314 (7). The main position of the disordered nitro group is close to the acetylamino group. The two aromatic rings are essentially coplanar, although they have a dihedral angle of 6.6 (5) °. In the *N*,*N*-diethylamino group at 4-positon, two ethyl chains tend to stand on the same side of the aminobenzene plane. The intramolecular N—H···N hydrogen bond links the amino and azo groups. (Table 1).

### Experimental

The crystals were obtained by dissolving 0.1 g of the title compound in 20 ml of acetone at room temperature and the resulting solution was covered with Parafilm plastic containing pin holes for slow evaporation of the solvent.

### Refinement

The nitro group at 2'-position and the cyano group at 6'-position are disordered over two sites with a refined site-occupancy factors of 0.686 (7): 0.314 (7). All hydrogen atoms were placed in their calculated positions, with N—H = 0.86 Å, and C—H = 0.93 or 0.96 Å, and refined using a riding model, with *U*_iso_ = 1.2 *U*_eq_ (N) and *U*_iso_ = 1.2 or 1.5 *U*_eq_ (C).

### Crystal data

**Table:**

C_19_H_19_N_7_O_5_	*F*(000) = 888
*M**_r_* = 425.41	*D*_x_ = 1.417 Mg m^−^^3^
Monoclinic, *P*2_1_/*c*	Mo *K*α radiation, λ = 0.71073 Å
Hall symbol: -P 2ybc	Cell parameters from 1060 reflections
*a* = 5.0995 (5) Å	θ = 2.7–26.4°
*b* = 30.792 (3) Å	µ = 0.11 mm^−^^1^
*c* = 12.7211 (11) Å	*T* = 298 K
β = 93.569 (1)°	Prism, blue
*V* = 1993.6 (3) Å^3^	0.37 × 0.11 × 0.07 mm
*Z* = 4	

### Data collection

**Table:**

Bruker SMART CCD area-detector diffractometer	3487 independent reflections
Radiation source: fine-focus sealed tube	1453 reflections with *I* > 2σ(*I*)
Graphite monochromator	*R*_int_ = 0.108
φ and ω scans	θ_max_ = 25.0°, θ_min_ = 2.6°
Absorption correction: multi-scan (*SADABS*; Sheldrick, 1996)	*h* = −6→6
*T*_min_ = 0.962, *T*_max_ = 0.993	*k* = −35→36
10384 measured reflections	*l* = −15→12

### Refinement

**Table:**

Refinement on *F*^2^	Primary atom site location: structure-invariant direct methods
Least-squares matrix: full	Secondary atom site location: difference Fourier map
*R*[*F*^2^ > 2σ(*F*^2^)] = 0.067	Hydrogen site location: inferred from neighbouring sites
*wR*(*F*^2^) = 0.157	H-atom parameters constrained
*S* = 1.00	*w* = 1/[σ^2^(*F*_o_^2^) + (0.0376*P*)^2^] where *P* = (*F*_o_^2^ + 2*F*_c_^2^)/3
3487 reflections	(Δ/σ)_max_ = 0.001
311 parameters	Δρ_max_ = 0.20 e Å^−^^3^
0 restraints	Δρ_min_ = −0.19 e Å^−^^3^

### Special details

**Table:**

Geometry. All s.u.'s (except the s.u. in the dihedral angle between two l.s. planes) are estimated using the full covariance matrix. The cell s.u.'s are taken into account individually in the estimation of s.u.'s in distances, angles and torsion angles; correlations between s.u.'s in cell parameters are only used when they are defined by crystal symmetry. An approximate (isotropic) treatment of cell s.u.'s is used for estimating s.u.'s involving l.s. planes.
Refinement. Refinement of *F*^2^ against ALL reflections. The weighted *R*-factor *wR* and goodness of fit *S* are based on *F*^2^, conventional *R*-factors *R* are based on *F*, with *F* set to zero for negative *F*^2^. The threshold expression of *F*^2^ > σ(*F*^2^) is used only for calculating *R*-factors(gt) *etc*. and is not relevant to the choice of reflections for refinement. *R*-factors based on *F*^2^ are statistically about twice as large as those based on *F*, and *R*- factors based on ALL data will be even larger.

### Fractional atomic coordinates and isotropic or equivalent isotropic displacement parameters (Å^2^)

**Table:**

	*x*	*y*	*z*	*U*_iso_*/*U*_eq_	Occ. (<1)
N1	0.4053 (6)	0.60751 (10)	0.4507 (2)	0.0579 (9)	
N2	0.5520 (6)	0.63947 (10)	0.4218 (2)	0.0576 (9)	
N3	0.3410 (6)	0.69052 (10)	0.5644 (2)	0.0574 (9)	
H3	0.4695	0.6863	0.5249	0.069*	
N4	−0.2983 (6)	0.61740 (10)	0.7645 (2)	0.0557 (9)	
N5	0.8292 (11)	0.70338 (16)	0.3319 (4)	0.0671 (14)	0.686 (7)
C19'	0.8292 (11)	0.70338 (16)	0.3319 (4)	0.0671 (14)	0.314 (7)
N6	1.2621 (8)	0.59919 (14)	0.1118 (3)	0.0658 (10)	
N7	0.507 (2)	0.5147 (3)	0.3559 (8)	0.091 (3)	0.686 (7)
N7'	0.861 (6)	0.7323 (10)	0.367 (2)	0.100 (8)	0.314 (7)
O1	0.1396 (6)	0.74446 (9)	0.6539 (2)	0.0863 (10)	
O2	0.6223 (11)	0.72310 (15)	0.3001 (4)	0.0819 (19)	0.686 (7)
O3	0.9997 (16)	0.7223 (4)	0.3842 (9)	0.096 (3)	0.686 (7)
O2'	0.410 (2)	0.5311 (5)	0.2877 (9)	0.079 (4)	0.314 (7)
O3'	0.697 (3)	0.5215 (4)	0.4102 (9)	0.089 (4)	0.314 (7)
O4	1.4090 (6)	0.62839 (10)	0.0872 (2)	0.0829 (10)	
O5	1.2586 (7)	0.56315 (12)	0.0723 (3)	0.1043 (12)	
C1	0.2380 (8)	0.61505 (12)	0.5298 (3)	0.0485 (10)	
C2	0.1973 (7)	0.65404 (12)	0.5871 (3)	0.0500 (10)	
C3	0.0152 (8)	0.65415 (13)	0.6638 (3)	0.0567 (11)	
H3A	−0.0135	0.6797	0.7003	0.068*	
C4	−0.1276 (8)	0.61654 (13)	0.6879 (3)	0.0526 (10)	
C5	−0.0889 (8)	0.57806 (13)	0.6280 (3)	0.0596 (11)	
H5	−0.1853	0.5531	0.6401	0.071*	
C6	0.0887 (8)	0.57827 (13)	0.5538 (3)	0.0583 (11)	
H6	0.1140	0.5528	0.5165	0.070*	
C7	0.3066 (9)	0.73243 (14)	0.5963 (4)	0.0672 (12)	
C8	0.4882 (9)	0.76442 (13)	0.5484 (4)	0.0805 (14)	
H8A	0.4524	0.7652	0.4735	0.121*	
H8B	0.6671	0.7557	0.5642	0.121*	
H8C	0.4608	0.7928	0.5771	0.121*	
C9	−0.4391 (8)	0.57792 (14)	0.7952 (3)	0.0706 (13)	
H9A	−0.4896	0.5615	0.7321	0.085*	
H9B	−0.5990	0.5866	0.8270	0.085*	
C10	−0.2868 (11)	0.54915 (15)	0.8696 (4)	0.0994 (17)	
H10A	−0.1265	0.5407	0.8395	0.149*	
H10B	−0.3886	0.5238	0.8831	0.149*	
H10C	−0.2465	0.5644	0.9345	0.149*	
C11	−0.3232 (8)	0.65583 (13)	0.8310 (3)	0.0612 (11)	
H11A	−0.4893	0.6544	0.8640	0.073*	
H11B	−0.3265	0.6815	0.7868	0.073*	
C12	−0.1039 (9)	0.66039 (14)	0.9157 (3)	0.0769 (13)	
H12A	−0.1042	0.6357	0.9618	0.115*	
H12B	−0.1297	0.6864	0.9554	0.115*	
H12C	0.0614	0.6620	0.8837	0.115*	
C13	0.7156 (8)	0.62571 (13)	0.3432 (3)	0.0499 (10)	
C14	0.8682 (8)	0.65840 (13)	0.3000 (3)	0.0521 (10)	
C15	1.0444 (8)	0.65038 (14)	0.2260 (3)	0.0585 (11)	
H15	1.1451	0.6728	0.2006	0.070*	
C16	1.0710 (8)	0.60897 (13)	0.1897 (3)	0.0549 (11)	
C17	0.9265 (8)	0.57544 (14)	0.2275 (3)	0.0620 (12)	
H17	0.9479	0.5473	0.2026	0.074*	
C18	0.7464 (8)	0.58378 (13)	0.3040 (3)	0.0539 (11)	
C19	0.6071 (11)	0.54535 (17)	0.3394 (4)	0.0660 (13)	0.686 (7)
N5'	0.6071 (11)	0.54535 (17)	0.3394 (4)	0.0660 (13)	0.314 (7)

### Atomic displacement parameters (Å^2^)

**Table:**

	*U*^11^	*U*^22^	*U*^33^	*U*^12^	*U*^13^	*U*^23^
N1	0.047 (2)	0.064 (2)	0.062 (2)	−0.0022 (18)	0.0040 (19)	0.0079 (17)
N2	0.049 (2)	0.057 (2)	0.067 (2)	−0.0032 (18)	0.0078 (18)	0.0001 (17)
N3	0.044 (2)	0.055 (2)	0.076 (2)	0.0024 (17)	0.0234 (18)	−0.0031 (17)
N4	0.040 (2)	0.058 (2)	0.071 (2)	−0.0015 (17)	0.0138 (18)	0.0028 (18)
N5	0.064 (4)	0.064 (3)	0.074 (3)	−0.009 (3)	0.017 (3)	−0.010 (2)
C19'	0.064 (4)	0.064 (3)	0.074 (3)	−0.009 (3)	0.017 (3)	−0.010 (2)
N6	0.063 (3)	0.073 (3)	0.063 (3)	0.014 (2)	0.020 (2)	−0.001 (2)
N7	0.102 (8)	0.076 (6)	0.098 (7)	−0.011 (6)	0.025 (6)	−0.007 (5)
N7'	0.11 (3)	0.087 (18)	0.106 (17)	0.003 (16)	0.014 (19)	−0.014 (12)
O1	0.073 (2)	0.068 (2)	0.123 (3)	−0.0030 (17)	0.052 (2)	−0.0156 (18)
O2	0.067 (4)	0.067 (3)	0.112 (4)	0.025 (3)	0.009 (3)	0.003 (3)
O3	0.078 (7)	0.091 (6)	0.116 (5)	0.001 (5)	−0.021 (5)	−0.022 (4)
O2'	0.066 (9)	0.094 (9)	0.075 (9)	−0.006 (7)	−0.013 (6)	−0.001 (7)
O3'	0.088 (11)	0.080 (8)	0.097 (10)	0.002 (7)	−0.001 (7)	0.035 (7)
O4	0.066 (2)	0.088 (2)	0.099 (2)	−0.0012 (18)	0.0371 (19)	0.0119 (18)
O5	0.112 (3)	0.088 (3)	0.120 (3)	0.001 (2)	0.060 (2)	−0.023 (2)
C1	0.041 (3)	0.054 (2)	0.052 (3)	0.004 (2)	0.011 (2)	0.001 (2)
C2	0.036 (3)	0.053 (3)	0.062 (3)	−0.004 (2)	0.007 (2)	0.007 (2)
C3	0.046 (3)	0.059 (3)	0.066 (3)	0.004 (2)	0.009 (2)	0.003 (2)
C4	0.037 (3)	0.060 (3)	0.061 (3)	−0.001 (2)	0.006 (2)	0.003 (2)
C5	0.051 (3)	0.062 (3)	0.067 (3)	−0.010 (2)	0.014 (2)	0.002 (2)
C6	0.054 (3)	0.058 (3)	0.064 (3)	−0.003 (2)	0.008 (2)	0.001 (2)
C7	0.054 (3)	0.064 (3)	0.085 (3)	−0.005 (3)	0.019 (3)	−0.006 (2)
C8	0.069 (3)	0.067 (3)	0.109 (4)	−0.012 (3)	0.037 (3)	−0.004 (2)
C9	0.053 (3)	0.074 (3)	0.088 (3)	−0.008 (2)	0.027 (3)	0.001 (3)
C10	0.122 (5)	0.080 (4)	0.097 (4)	−0.006 (3)	0.015 (3)	0.019 (3)
C11	0.045 (3)	0.061 (3)	0.080 (3)	0.005 (2)	0.025 (2)	−0.002 (2)
C12	0.060 (3)	0.084 (3)	0.086 (3)	0.004 (3)	0.005 (3)	−0.016 (2)
C13	0.045 (3)	0.057 (3)	0.048 (3)	0.004 (2)	0.004 (2)	−0.005 (2)
C14	0.043 (3)	0.057 (3)	0.057 (3)	0.003 (2)	0.007 (2)	−0.004 (2)
C15	0.048 (3)	0.068 (3)	0.060 (3)	−0.001 (2)	0.008 (2)	0.004 (2)
C16	0.052 (3)	0.058 (3)	0.057 (3)	0.001 (2)	0.012 (2)	0.000 (2)
C17	0.056 (3)	0.065 (3)	0.066 (3)	0.003 (2)	0.011 (2)	−0.004 (2)
C18	0.046 (3)	0.059 (3)	0.057 (3)	−0.005 (2)	0.006 (2)	0.007 (2)
C19	0.059 (4)	0.069 (3)	0.070 (4)	0.000 (3)	0.013 (3)	0.000 (3)
N5'	0.059 (4)	0.069 (3)	0.070 (4)	0.000 (3)	0.013 (3)	0.000 (3)

### Geometric parameters (Å, º)

**Table:**

N1—N2	1.303 (4)	C7—C8	1.506 (5)
N1—C1	1.379 (4)	C8—H8A	0.9600
N2—C13	1.407 (4)	C8—H8B	0.9600
N3—C7	1.367 (5)	C8—H8C	0.9600
N3—C2	1.382 (4)	C9—C10	1.481 (6)
N3—H3	0.8600	C9—H9A	0.9700
N4—C4	1.347 (4)	C9—H9B	0.9700
N4—C11	1.465 (4)	C10—H10A	0.9600
N4—C9	1.477 (5)	C10—H10B	0.9600
N5—O3	1.211 (12)	C10—H10C	0.9600
N5—O2	1.262 (7)	C11—C12	1.510 (5)
N5—C14	1.460 (6)	C11—H11A	0.9700
N6—O5	1.218 (4)	C11—H11B	0.9700
N6—O4	1.224 (4)	C12—H12A	0.9600
N6—C16	1.464 (5)	C12—H12B	0.9600
N7—C19	1.099 (8)	C12—H12C	0.9600
O1—C7	1.215 (4)	C13—C18	1.396 (5)
C1—C6	1.409 (5)	C13—C14	1.405 (5)
C1—C2	1.426 (5)	C14—C15	1.364 (5)
C2—C3	1.388 (5)	C15—C16	1.366 (5)
C3—C4	1.412 (5)	C15—H15	0.9300
C3—H3A	0.9300	C16—C17	1.373 (5)
C4—C5	1.429 (5)	C17—C18	1.403 (5)
C5—C6	1.349 (5)	C17—H17	0.9300
C5—H5	0.9300	C18—C19	1.465 (6)
C6—H6	0.9300		
			
N2—N1—C1	118.1 (3)	N4—C9—H9A	108.6
N1—N2—C13	110.4 (3)	C10—C9—H9A	108.6
C7—N3—C2	128.8 (3)	N4—C9—H9B	108.6
C7—N3—H3	115.6	C10—C9—H9B	108.6
C2—N3—H3	115.6	H9A—C9—H9B	107.6
C4—N4—C11	121.1 (3)	C9—C10—H10A	109.5
C4—N4—C9	121.4 (3)	C9—C10—H10B	109.5
C11—N4—C9	116.8 (3)	H10A—C10—H10B	109.5
O3—N5—O2	120.2 (8)	C9—C10—H10C	109.5
O3—N5—C14	120.2 (7)	H10A—C10—H10C	109.5
O2—N5—C14	119.5 (5)	H10B—C10—H10C	109.5
O5—N6—O4	123.9 (4)	N4—C11—C12	113.6 (3)
O5—N6—C16	118.3 (4)	N4—C11—H11A	108.8
O4—N6—C16	117.8 (4)	C12—C11—H11A	108.8
N1—C1—C6	113.0 (3)	N4—C11—H11B	108.8
N1—C1—C2	129.1 (3)	C12—C11—H11B	108.8
C6—C1—C2	117.9 (4)	H11A—C11—H11B	107.7
N3—C2—C3	122.1 (3)	C11—C12—H12A	109.5
N3—C2—C1	118.8 (3)	C11—C12—H12B	109.5
C3—C2—C1	119.1 (3)	H12A—C12—H12B	109.5
C2—C3—C4	121.8 (4)	C11—C12—H12C	109.5
C2—C3—H3A	119.1	H12A—C12—H12C	109.5
C4—C3—H3A	119.1	H12B—C12—H12C	109.5
N4—C4—C3	120.4 (4)	C18—C13—C14	116.4 (4)
N4—C4—C5	121.3 (4)	C18—C13—N2	128.1 (4)
C3—C4—C5	118.3 (4)	C14—C13—N2	115.5 (3)
C6—C5—C4	119.4 (4)	C15—C14—C13	123.1 (4)
C6—C5—H5	120.3	C15—C14—N5	118.1 (4)
C4—C5—H5	120.3	C13—C14—N5	118.8 (4)
C5—C6—C1	123.4 (4)	C14—C15—C16	119.0 (4)
C5—C6—H6	118.3	C14—C15—H15	120.5
C1—C6—H6	118.3	C16—C15—H15	120.5
O1—C7—N3	125.0 (4)	C15—C16—C17	121.1 (4)
O1—C7—C8	121.1 (4)	C15—C16—N6	120.3 (4)
N3—C7—C8	113.8 (4)	C17—C16—N6	118.5 (4)
C7—C8—H8A	109.5	C16—C17—C18	119.6 (4)
C7—C8—H8B	109.5	C16—C17—H17	120.2
H8A—C8—H8B	109.5	C18—C17—H17	120.2
C7—C8—H8C	109.5	C13—C18—C17	120.7 (4)
H8A—C8—H8C	109.5	C13—C18—C19	124.7 (4)
H8B—C8—H8C	109.5	C17—C18—C19	114.6 (4)
N4—C9—C10	114.7 (4)	N7—C19—C18	172.6 (7)
			
C1—N1—N2—C13	178.1 (3)	C9—N4—C11—C12	94.0 (4)
N2—N1—C1—C6	179.7 (3)	N1—N2—C13—C18	−5.6 (5)
N2—N1—C1—C2	0.4 (6)	N1—N2—C13—C14	175.2 (3)
C7—N3—C2—C3	−11.5 (6)	C18—C13—C14—C15	−2.0 (6)
C7—N3—C2—C1	168.6 (4)	N2—C13—C14—C15	177.4 (4)
N1—C1—C2—N3	−0.5 (6)	C18—C13—C14—N5	175.3 (4)
C6—C1—C2—N3	−179.7 (4)	N2—C13—C14—N5	−5.4 (6)
N1—C1—C2—C3	179.6 (4)	O3—N5—C14—C15	−70.7 (8)
C6—C1—C2—C3	0.4 (5)	O2—N5—C14—C15	105.7 (5)
N3—C2—C3—C4	−178.8 (3)	O3—N5—C14—C13	111.9 (7)
C1—C2—C3—C4	1.1 (6)	O2—N5—C14—C13	−71.7 (6)
C11—N4—C4—C3	−5.6 (6)	C13—C14—C15—C16	1.7 (6)
C9—N4—C4—C3	−176.6 (4)	N5—C14—C15—C16	−175.6 (4)
C11—N4—C4—C5	175.4 (4)	C14—C15—C16—C17	−1.0 (6)
C9—N4—C4—C5	4.4 (6)	C14—C15—C16—N6	−178.8 (4)
C2—C3—C4—N4	178.4 (4)	O5—N6—C16—C15	−171.5 (4)
C2—C3—C4—C5	−2.6 (6)	O4—N6—C16—C15	5.6 (6)
N4—C4—C5—C6	−178.4 (4)	O5—N6—C16—C17	10.6 (6)
C3—C4—C5—C6	2.6 (6)	O4—N6—C16—C17	−172.3 (4)
C4—C5—C6—C1	−1.2 (6)	C15—C16—C17—C18	0.6 (6)
N1—C1—C6—C5	−179.6 (4)	N6—C16—C17—C18	178.5 (4)
C2—C1—C6—C5	−0.3 (6)	C14—C13—C18—C17	1.6 (6)
C2—N3—C7—O1	1.6 (7)	N2—C13—C18—C17	−177.7 (4)
C2—N3—C7—C8	−175.1 (4)	C14—C13—C18—C19	179.5 (4)
C4—N4—C9—C10	82.3 (5)	N2—C13—C18—C19	0.3 (7)
C11—N4—C9—C10	−89.0 (5)	C16—C17—C18—C13	−1.0 (6)
C4—N4—C11—C12	−77.3 (5)	C16—C17—C18—C19	−179.1 (4)

### Hydrogen-bond geometry (Å, º)

**Table:**

*D*—H···*A*	*D*—H	H···*A*	*D*···*A*	*D*—H···*A*
N3—H3···N2	0.86	2.01	2.676 (4)	133
